# Changing Incidence of Inflammatory Bowel Disease: Environmental Influences and Lessons Learnt from the South Asian Population

**DOI:** 10.3389/fped.2013.00034

**Published:** 2013-11-06

**Authors:** Alice Foster, Kevan Jacobson

**Affiliations:** ^1^Division of Gastroenterology, Hepatology and Nutrition, University of British Columbia, Vancouver, BC, Canada; ^2^Child and Family Research Institute, University of British Columbia, Vancouver, BC, Canada; ^3^Department of Cellular and Physiological Sciences, BC Children’s Hospital, University of British Columbia, Vancouver, BC, Canada

**Keywords:** inflammatory bowel disease, environmental factors, South Asian population

## Abstract

Inflammatory bowel disease (IBD) is a chronic relapsing inflammatory disorder of the gastrointestinal tract associated with significant morbidity. While IBD occurs in genetically susceptible individuals, the etiology is multifactorial, involving environmental influences, intestinal dysbiosis, and altered immune responses. The rising incidence of IBD in industrialized countries and the emergence of IBD in countries with traditionally low prevalence underscore the importance of environmental influences in the pathobiology of the disease. Moreover the high incidence of IBD observed in the South Asian immigrant population in the United Kingdom and Canada further supports the influence of environmental factors.

## Introduction

Inflammatory bowel disease (IBD) is a chronic relapsing inflammatory condition of the gastrointestinal tract that includes both Crohn’s disease (CD) and ulcerative colitis (UC). Since the identification of CD by Crohn (1) and UC by various physicians in the latter half of the nineteenth century, IBD has been increasingly identified in populations of northern and western Europe, North America, and Australia (1, 2). More recently IBD has developed a wider geographic distribution, emerging in populations previously thought to be at low risk. Two recent comprehensive reviews of temporal trends in worldwide incidence rates of pediatric and adult IBD highlight the rising global rates in both children and adults in both developed and developing nations (3, 4). Furthermore, studies have shown that individuals who migrate from low prevalent areas (e.g., South Asia) to high prevalent countries (e.g., Canada and England) are at increased risk for developing IBD, particularly among first- and second-generation immigrants highlighting the importance of environmental influences (5–7). With increasing worldwide prevalence, IBD is becoming a significant societal burden, as it generally requires lifelong therapy and monitoring.

Inflammatory bowel disease is a challenging disease as the etiology remains unclear, the disease severity is variable and unpredictable, and treatment options are still quite limited and focused on induction and maintenance of remission and not cure. IBD is considered to be a multifactorial disease arising from the combined effects of genetic susceptibility, environmental influences, intestinal dysbiosis, and variation of innate and adaptive immune responses that trigger an inflammatory response. It is believed that each of these factors play a contributing role in the induction of disease and influence the disease course, with varying levels of influence occurring in pediatrics and adults and in different population groups.

The South Asian immigrant population in the United Kingdom (UK) and in Canada has demonstrated higher rates of IBD than in the indigenous populations and in the population in their countries of origin, reinforcing the importance of environmental influences (7–10). In this article, we review the literature regarding those factors implicated in the pathogenesis of IBD with particular attention to the South Asian populations.

## Incidence and Prevalence Data

Epidemiological studies to date examining the South Asian population are limited to single urban regions that include patients with access to healthcare (11, 12). In India, adult UC prevalence is reported as 44.3 cases per 100,000 inhabitants and the crude incidence is reported as 6.02 cases per 100,000 inhabitants per year (11). Comparisons between different countries’ data suggest that the adult IBD incidence and prevalence is significantly lower in the South Asian population than in North America or the UK which has reported prevalence rates of 144–243 cases per 100,000 inhabitants, and incidence rates of 7.9–20.2 cases per 100,000 inhabitants per year (13–15). Interestingly, small single center studies in England suggest that IBD is seen with greater frequency in patients from South Asia who subsequently migrate to the UK (Table 1) (8). Similarly in the pediatric IBD population of British Columbia, Canada, a higher IBD incidence rate has been reported in the South Asian community than in other ethnicities, including children of Western European descent (Table 1) (7). These studies suggest that environmental factors are influential in IBD pathogenesis when an underlying genetic predisposition is present.

**Table 1:** **Characteristics of South Asian immigrant studies**.

Study	Disease	Population	Location	Years	Incidence in South Asian population (per 100,000)	Incidence in non-South Asian population (per 100,000)
Pinsk et al. (7)	UC and CD	Pediatric	British Columbia, Canada	1996–2001	15.19	5.19
Fellows et al. (8)	CD	Adult and Pediatric	Derby, UK	1966–1985	4.39	7.47
Probert et al. (9)	UC	Adult and Pediatric	Leicestershire, UK	1972–1980	9.3	3.6
				1981–1989	10.8	5.3
Jayanthi et al. (10)	CD	Adult and Pediatric	Leicestershire, UK	1972–1980	1.24	3.47
				1981–1989	3.1	5.27

Ref. (7) of the total population 84% second generation; 84% of families originating from the Punjab, 79% Sikh, 11% Muslim, 4% Hindu.

Ref. (9) of the total population 78% first generation, all South Asian children with UC born in the UK, annual UC incidence × 10^5^ Sikh, Hindu, and Muslim 1972–80/1981–89: 0/16.5, 13.4/10.8, 4.6/6.2, respectively, Hindus and Sikhs had significantly more UC than Europeans.

Ref. (10) annual CD incidence × 10^5^ Sikh, Hindu, and Muslim 1972–80/1981–89: 1.8/3.4, 1.4/2.4, 0.9/5.4, respectively.

## Environment

The relationship between the environment and pathogenesis of IBD remains difficult to define in part due to the effect of confounding variables and timing of exposure(s), which likely occurs during vulnerable periods of development and may precede onset of the disease by years.

## Diet

Immigration to a western country is associated with experiences that impact significantly on dietary choices. High costs along with limited accessibility and availability of ingredients used in the preparation of traditional diets can result in alterations in the diet (16). Immigrant families also frequently modify their diet to assimilate with the indigenous population, adopting both their food choices and eating patterns, referred to as “dietary acculturation” (16).

Specifically, dietary changes are noticeable in the South Asian population after emigration to a Western country. The traditional South Asian diet consists largely of carbohydrate-rich foods including wheat, rice, sorghum, and pearl millet, with minimal consumption of fat-rich foods and sugary drinks (17). A low meat, fish, and dairy diet are influenced both by the countries high levels of poverty, along with religious and cultural beliefs (18). In contrast, Western countries have exposed the South Asian population to a more processed diet with reduced nutritional quality including foods higher in fats, sugars, and refined carbohydrates. This had led to decreased intake of healthy foods such and fruits, vegetables, whole grains, and fiber, resulting in decreased consumption of trace minerals, vitamins, antioxidants, and essential polyunsaturated fatty acids (PUFA). Fiber intake is important as it decreases serum lipids, improves glycemic control and has anti-inflammatory properties (18). A previous Japanese study reported a more than a twofold increased risk of CD following similar dietary changes characterized by consumption of diets high in fats and oils, sugars, sweeteners, and sweets (19). A recent systematic review also reported increased risk of developing CD with high intake of PUFAs, omega-6-fatty acids, saturated fats and meat, and increased risk of UC with high intake of total fat, PUFAs, omega-6 fatty acids, and meat (20). On the contrary, high intake of fiber and fruits was negatively associated with CD risk (20).

A few small studies have supported that a dietary change occurs with immigration to the western world. A Norwegian study demonstrated that 45% of Pakistani and 63% of Sri Lankan immigrants had an increase in meat intake, and 30 and 45%, respectively reported a decrease in beans and lentils (21). Increased intake of high fat dairy products has also been observed in Pakistani women immigrating to Norway, together with increased consumption of *n*−6 PUFA and decreased intake of *n*−3 PUFA (22). South Asian immigrants were also found to have a decreased intake of *n*−3 PUFA and an increase in *n*−6 PUFA consumption (23, 24).

A similar dietary change has been observed in pediatric patients in the UK in which South Asian immigrants were found to have higher energy and fat intake and lower carbohydrate intake than white European children (25). Similarly, the South Asian pediatric IBD population of British Columbia, Canada was found to have a diet high in fat, refined sugar, and low in fresh fruits and vegetables compared with their parents and grandparents (7). In this study, 84% of South Asian IBD patients were second-generation immigrants, reinforcing the link between Westernization of diets amongst the immigrant populations and the changing incidence of IBD.

Experimental data support the link between changing dietary intake of PUFAs and development of IBD in genetically susceptible individuals (26–29). Indeed, increased intake of omega-6-fatty acids results in increased synthesis and membrane incorporation of arachidonic acid (ARA) with the production of pro-inflammatory mediators, and increased oxidative stress in *n*−6 fatty acid rich membranes. In contrast, the *n*−3 PUFAs eicosapentaenoic acid (EPA) and docosahexaenoic acid (DHA) partly replace arachidonic acid in cell membranes and reduce inflammation.

Another dietary shift occurring in the South Asian population is a reduction in fermented food consumption. In India, fermented foods have been an integral component of the diet for centuries (30). With limited availability to refrigerate foods, fermentation was a technique developed using lactic acid bacteria to help with food preservation. Consumption of the food and associated bacteria are believed to alter the composition of the intestinal microbiota (31). The bacteria are referred to as probiotics, and are defined by the World Health Organization as “live microorganisms that, when administered in adequate amounts, confer a health benefit on the consumer.” [FAO/WHO Guidelines for the evaluation of probiotics in food (London, ON, Canada) 2002, http://www.fao.org/es/ESN/Probio/probio.htm]. Probiotics are believed to modulate the immune system and improve the function of the intestinal mucosal barrier (31). The integrity of this mucosal barrier is thought to play an important role in IBD pathogenesis (32), and as such the role of probiotics must be considered, and is an area needing ongoing research. With increased availability of refrigeration in South Asia, and across industrialized nations, consumption of fermented foods has likely decreased in indigenous and immigrant populations, potentially influencing the IBD prevalence rates in this subgroup.

Breastfeeding is another possible dietary factor that may be influential in IBD development. A recent meta-analysis analyzed 17 studies addressing the hypothesis that breastfeeding is a protective factor against IBD noted that the limited data supports the hypothesis that breastfeeding confers protection, however larger more well organized studies are needed (33). Developed countries likely have easier access to infant formulas, and as such may be a factor in rising rates of IBD in the Western world. A British study focusing on a group of ethnic minority women, exploring factors impacting breastfeeding decisions, reported in particular that women from the South Asian population indicated that barriers to breastfeeding included lack of privacy associated with co-inhabitation with a large extended family, and grandparental pressure (34). Living situations following emigration may differ from those in their native country, and as such may account for changes in both initiation and maintenance of breastfeeding. Indeed, the Canadian pediatric IBD study reported that 69% of children with IBD were breast fed (56% for more than 6-months), in contrast to 92% of parents (80% breast fed for more than 6-months) (7). A confounding variable is the changing maternal dietary fat intake and the impact on the offspring. The developing fetus and young breast-fed infant is solely dependent on the mother for an adequate supply of essential *n*−6 and *n*−3 fatty acids. Placental transfer and secretion of *n*−6 and *n*−3 fatty acids into breast milk is variable and dependent on maternal dietary intake of *n*−6 and *n*−3 fatty acids, which in turn influences plasma membrane phospholipids and inflammatory potential of the offspring (35–38).

While, dietary studies to date have shown inconsistent findings and have been limited by study design and sample size, dietary changes observed in IBD patients in industrialized countries are strikingly similar to changes observed in immigrant populations and within South Asia (18) and thus remain an important variable in the pathogenesis and changing incidence of IBD. We can expect that IBD incidence rates will continue rise in countries with such dietary shifts. However, large population based-studies are required both in developed and in developing countries such as South Asia to provide more conclusive support for the link between changing diet and rising incidence of IBD.

## The Urban Environment

Inflammatory bowel disease incidence rates are higher in the more industrialized regions of North America and Europe, reinforcing the important influence of the urban environment in contributing to IBD pathogenesis. A recent large systematic review and meta analysis of 40 studies showed that living in an urban environment may increase the risk of development of UC and CD with a pooled incidence rate ratio (IRR) of 1.17 (95% CI 1.03–1.32) for UC and 1.42 (95% CI 1.26–1.60) for CD (4).

A number of confounding variables must be taken into account, and as mentioned previously the role of dietary changes needs to be considered. A cross sectional study in India comparing the diets of over 6500 individuals living in both urban and rural communities reported that those living in an urban setting had a higher caloric intake, and specifically a higher intake of protein, fat, and carbohydrates (39).

Other considerations associated with urban living include population density, education, lifestyle changes, and exposure to industrial pollutants (40). To date, studies examining the relationship between air pollution within the urban setting and disease have primarily focused on its effects on the respiratory and cardiovascular systems rather than the effects of pollutants on the intestinal tract. A database study from the UK found that exposure to sulfur dioxide and nitrogen dioxide may increase the risk of early onset UC and CD, respectively (41). An ecological study found a strong correlation between total air emissions of criteria pollutants and hospitalizations in adults with IBD (42). As the studies have been limited, further investigation is warranted to increase our understanding of the role of air pollutants in IBD pathogenesis.

The urban environments typically have improved sanitation and hygiene practices than a rural setting, likely altering and restricting exposure to bacteria in early life. Strachan proposed the “hygiene hypothesis,” suggesting that exposure to common infections during immune system development help prepare for immune challenges later in life, reducing the likelihood of developing immune mediated/inflammatory disease (43). This hypothesis is consistent with evidence that IBD is more prevalent in areas of the world with less infections, improved sanitary living conditions, wide spread vaccine and antibiotic use, and clean food and water (44, 45). Additional research has suggested that lower rates of IBD may be associated with populations exposed to higher pathogen burdens, those living on farms, drinking unpasteurized milk, exposure to household pets, and having a larger family size (46). However it remains unclear whether this lack of exposure to pathogens at an early age does result in an inappropriate immune response and development of IBD in life (47).

When evaluating rural and urban influences on IBD prevalence the validity of the available data needs to be considered. Most data collection occurs in cities with larger hospitals where the studied population has better access to healthcare and the medical providers may have more exposure and heightened awareness of a disease. In contrast, the data from rural regions may have more inaccuracies as IBD may go unrecognized and/or access to medical care may be limited.

## Vitamin D

Northern communities of Europe and North America have high rates of vitamin D deficiency correlating with higher rates of IBD (48). In contrast, lower rates of IBD are observed in those living closer to the equator (49). Indeed, one would anticipate that the South Asian population migrating to the Northern communities of Europe and North America would be at higher risk of vitamin D deficiency (49), an additional factor that must be considered to explain the rising incidence of IBD in this population.

Both adult and pediatric studies demonstrate that IBD patients are often deficient in vitamin D (50, 51). Moreover, a recent study in British Columbia, Canada reported that South Asian adult IBD patients had consistently lower average serum Vitamin D levels than non-South Asian patients (average 25OH vitamin D level ±SD (nanomoles per liter) South Asian vs. non-South Asian 44.8 ± 18.1 vs. 66.4 ± 25.7, respectively) (52).

The link between IBD and vitamin D has been reinforced by animal models evaluating immune system modulation through the signaling of vitamin D through its receptor (VDR) (53). The VDR is expressed on monocytes, macrophages, and leukocytes (54, 55), dendritic cells (56, 57), activated CD4+ and CD8+ T cells (58), and intestinal epithelial cells (48). Vitamin D has been shown to inhibit antigen presenting cell production of IL-12, a cytokine responsible for T-helper cell development (59), and also to directly inhibit T-cell activation and proliferation (60). Additionally, vitamin D has been noted to induce expression of a CD susceptibility gene NOD2 in monocytic and epithelial cells (48). Indeed, IBD mouse models that are VDR deficient have poor regulation of the immune system and impaired intestinal epithelial barrier integrity resulting in severe gastrointestinal inflammation (53). Conversely, vitamin D supplementation has shown therapeutic benefit, attenuating spontaneous enterocolitis in the IL-10 knock out mouse, and reducing the relapse rate in adult patients with quiescent CD (61, 62).

## Genetics

Over recent years there have been considerable advancements toward understanding the role of genetics in IBD. At present, over 160 susceptibility loci/genes for CD and UC have been identified (63). Genetic factors account for 13.6% of variance in CD and 7.5% for UC (64). Of the identified loci, 110 are common to both CD and UC, and more loci have been discovered with IBD than any other complex disease (65). Although identification of these loci allows a clearer understanding of the disease, the gene products, function, and their role in pathogenesis remain unclear. To date a majority of the genetic research has focused on IBD populations in North America and Northern Europe. A better understanding of IBD associated genes and genetic variability among different ethnic groups may advance our understanding of the disease. Consequently, a need for expansion to include other ethnicities, and their associated IBD genes is an important area of research as it is anticipated that additional genes likely exist for these populations including the South Asian population (66).

## Helicobacter pylori

*Helicobacter pylori* is a bacterial pathogen associated with chronic active gastritis, peptic ulcer disease, and gastric cancer that can be eradicated with antimicrobial therapy. A recent meta-analysis reported that IBD is negatively associated with this infection, suggesting a possible protective benefit of colonization (67). The bacteria affects T-cell regulatory gene expression, and as a result induces an anti-inflammatory response (67). As *H. pylori* is associated with crowded living conditions and poor sanitary conditions (68), this suggests a possible protective mechanism in the South Asian population prior to emigration. Exposure to improved sanitation and higher rates of antibiotic treatment in their country of origin and following emigration, associated with reduced rates of infection and increased rates of eradiation may contribute to rising IBD rates.

## Helminths

The development of IBD has also been linked to reduced exposure to helminths, thought to be involved in immune regulation of intestinal microflora (69). The effect of helminths is likely due to their promotion of T-helper 2 cytokines that are protective and have an anti-inflammatory role (70). Given this mechanism, therapeutic benefits of helminths have been investigated. A randomized control trial reported improvement of disease activity scores following 12 weeks of ingestion of the helminth *Trichuris suis* (71). Lower rates of helminth infections are seen in developed countries that have higher rates of IBD (71), and may contribute to rising IBD trends as South Asians emigrate to areas with lower parasitic burdens.

## Oral Contraceptives

A meta-analysis assessing the risk of oral contraceptive use with the etiology of IBD has supported a positive association with the relative risk increasing for CD with longer medication exposure time (72). More recently two large prospective cohorts provided additional support reporting that the risk of CD was increased with oral contraceptive use (73). These findings are supported by the potential pro-inflammatory action of estrogens through effects on antibody production, T-cell function and on proliferation of monocytes/macrophages (74). In developing countries hormonal contraceptive therapy was found to be limited by knowledge deficits, limited accessibility, concern over side effects, and fear of infertility (75). However, higher oral contraceptive therapy use is likely to occur with urbanization and globalization of the South Asian population in India and following immigration to industrialized countries in part due to improved healthcare access.

## Intestinal Microbiota

There is increasing evidence of a bidirectional interaction between the host’s intestinal microflora, the intestinal epithelium, and mucosal immune system. The presence of a highly diverse and robust intestinal microbial ecosystem is associated with a healthy intestinal epithelial barrier and appropriately responsive mucosal immune system. In IBD, patient studies have demonstrated increased number of mucosal bacteria with a less diversified intestinal flora (76, 77). Animal models using Interleukin 10 deficient mice have also supported a role for intestinal microbiota in the pathogenesis of IBD (78–80).

Increased exposure to antibiotics alters the intestinal microflora (81). One study, evaluating 36 children with IBD compared to controls reported that antibiotic use was more common in the first year of life in those children with IBD (58%) than controls (39%) (82). Similarly, an adult study found higher rates of antibiotic use 2–5 years prior to diagnosis of IBD (83). Research within this area has been limited due to the difficulty in demonstrating a clear causal relationship as a result of the various confounding variables. However, increased antibiotic use has occurred in industrialized nations and likely occurs with urbanization and following emigration of South Asians to more developed countries.

Secondly, recent studies suggest that variation in intestinal microbiota is influenced by diet (84, 85). A comparison of the intestinal microbiota of children from Europe on a westernized diet, and children from rural Africa on a high fiber diet has shown differences suggesting that diet impacts gut microbiota (86). For example, diets high in protein and saturated fats were associated with *Bacteroides* in the intestinal microflora, while those high in carbohydrates and simple sugars were associated with *Prevotella* (87). Microbes in our diet have also been shown to affect our intestinal flora through horizontal transfer of genes encoding specific carbohydrate degrading enzymes (88). This demonstrates compensatory adaptation of our intestinal flora to catabolize new dietary carbohydrates (88).

Thirdly, enteric infections modulate the intestinal commensal microorganisms. Recent studies in mouse models showed that during *Salmonella*-induced gastroenteritis the population of commensal flora was reduced (89). This change in the flora resulted in the accumulation of metabolites such as sugars, that are usually metabolized by the microbiota (89).

Hence exposure to different diets, increased use of antibiotics and exposure to new enteric pathogens following urbanization and globalization and through emigration likely has modifying effects on the host commensal microbiota and through these actions may contribute to the rising incidence of IBD.

## Summary

While it is believed that the IBD rates in South Asia are rising and following a similar trend to that observed in the Western world 50 years ago, it is currently difficult to provide clear evidence, as the data is limited. This is confounded by cases of IBD in South Asia that may have been previously misclassified as infectious in etiology. Furthermore increasing awareness and knowledge of IBD in South Asia associated with increased access to health care will further complicate determination of incidence. Conversely, studies in the UK and Canada suggest that following immigration, the South Asian immigrant population and particularly the immigrant Sikh population is a risk. Plausible risk factors include changes in diet, environmental exposures, and changes in intestinal flora. Further studies in these areas are required, both in the countries of origin and in the countries that these groups migrate to.

## Future Directions

Further research comparing trends between developing and developed countries needs to be undertaken to gain more insight into the role of environmental factors in the pathogenesis of IBD. More specifically, studying the effects of urbanization and migration will likely help increase our understanding of the interactions between genetics, the environment, and the disease. Moreover, better categorization of our patients based on understanding the complicated mix of past and present environmental factors, with population specific genetic variations, may indeed lead us on a path to better understanding of disease pathogenesis and targeted therapeutic approaches in the future.

## Conflict of Interest Statement

The authors declare that the research was conducted in the absence of any commercial or financial relationships that could be construed as a potential conflict of interest.

## References

[1] Crohn BB Regional ileitis a pathologic and clinical entity. JAMA (1932) 99(16):1323 10.1001/jama.1932.02740680019005

[2] Kirsner JB Historical aspects of inflammatory bowel disease. J Clin Gastroenterol (1988) 10(3):286–97 10.1097/00004836-198806000-00012 2980764

[3] Benchimol EI Fortinsky KJ Gozdyra P Van den Heuvel M Van Limbergen J Griffiths AM Epidemiology of pediatric inflammatory bowel disease: a systematic review of international trends. Inflamm Bowel Dis (2011) 17(1):423–39 10.1002/ibd.21349 20564651

[4] Soon IS Molodecky NA Rabi DM Ghali WA Barkema HW Kaplan GG The relationship between urban environment and the inflammatory bowel diseases: a systematic review and meta-analysis. BMC Gastroenterol (2012) 12(1):51 10.1186/1471-230X-12-51 22624994 PMC3517531

[5] Carr I Mayberry JF The effects of migration on ulcerative colitis: a three-year prospective study among Europeans and first- and second- generation South Asians in Leicester (1991-1994). Am J Gastroenterol (1999) 94(10):2918–22 10.1016/S0002-9270(99)00494-3 10520845

[6] Tsironi E Feakins RM Probert CSJ Roberts CSJ Rampton DS Phil D Incidence of inflammatory bowel disease is rising and abdominal tuberculosis is falling in Bangladeshis in East London, United Kingdom. Am J Gastroenterol (2004) 99(9):1749–55 10.1111/j.1572-0241.2004.30445.x 15330914

[7] Pinsk V Lemberg DA Grewal K Barker CC Schreiber RA Jacobson K Inflammatory bowel disease in the South Asian pediatric population of British Columbia. Am J Gastroenterol (2007) 102(5):1077–83 10.1111/j.1572-0241.2007.01124.x 17378907

[8] Fellows IW Freeman JG Holmes GK Crohn’s disease in the city of Derby, 1951-85. Gut (1990) 31(11):1262–5 10.1136/gut.31.11.1262 2253910 PMC1378696

[9] Probert CS Jayanthi V Pinder D Wicks AC Mayberry JF Epidemiological study of ulcerative proctocolitis in Indian migrants and the indigenous population of Leicestershire. Gut (1992) 33(5):687–93 10.1136/gut.33.5.687 1307684 PMC1379303

[10] Jayanthi V Probert CS Pinder D Wicks AC Mayberry JF Epidemiology of Crohn’s disease in Indian migrants and the indigenous population in Leicestershire. Q J Med (1992) 82(298):125–38 1620813

[11] Sood A Incidence and prevalence of ulcerative colitis in Punjab, North India. Gut (2003) 52(11):1587–90 10.1136/gut.52.11.1587 14570727 PMC1773859

[12] Khosla SN Girdhar NK Lal S Mishra DS Epidemiology of ulcerative colitis in hospital and select general population of northern India. J Assoc Physicians India (1986) 34(6):405–7 3771475

[13] Rubin GP Hungin APS Kelly PJ Ling J Inflammatory bowel disease: epidemiology and management in an English general practice population. Aliment Pharmacol Ther (2000) 14(12):1553–9 10.1046/j.1365-2036.2000.00886.x 11121902

[14] Loftus CG Loftus EV Jr Harmsen WS Zinsmeister AR Tremaine WJ Melton LJ III Update on the incidence and prevalence of Crohn’s disease and ulcerative colitis in Olmsted County, Minnesota, 1940-2000. Inflamm Bowel Dis (2007) 13(3):254–61 10.1002/ibd.20029 17206702

[15] Bernstein CN Wajda A Svenson LW MacKenzie A Koehoorn M Jackson M The epidemiology of inflammatory bowel disease in Canada: a population-based study. Am J Gastroenterol (2006) 101(7):1559–68 10.1111/j.1572-0241.2006.00603.x 16863561

[16] Satia JA Dietary acculturation and the nutrition transition: an overview. Appl Physiol Nutr Metab (2010) 35(2):219–23 10.1139/H10-007 20383236

[17] Misra A Rastogi K Joshi SR Whole grains and health: perspective for Asian Indians. J Assoc Physicians India (2009) 57:155–62 19582985

[18] Holmboe-Ottesen G Wandel M Changes in dietary habits after migration and consequences for health: a focus on South Asians in Europe. Food Nutr Res (2012) 56:18891 10.3402/fnr.v56i0.18891 23139649 PMC3492807

[19] Sakamoto N Kono S Wakai K Fukuda Y Satomi M Shimoyama T Dietary risk factors for inflammatory bowel disease: a multicenter case-control study in Japan. Inflamm Bowel Dis (2005) 11(2):154–63 10.1097/00054725-200502000-00009 15677909

[20] Hou JK Abraham B El-Serag H Dietary intake and risk of developing inflammatory bowel disease: a systematic review of the literature. Am J Gastroenterol (2011) 106(4):563–73 10.1038/ajg.2011.44 21468064

[21] Wandel M Råberg M Kumar B Holmboe-Ottesen G Changes in food habits after migration among South Asians settled in Oslo: the effect of demographic, socio-economic and integration factors. Appetite (2008) 50(2–3):376–85 10.1016/j.appet.2007.09.003 17949850

[22] Mellin-Olsen T Wandel M Changes in food habits among Pakistani immigrant women in Oslo, Norway. Ethn Health (2005) 10(4):311–39 10.1080/13557850500145238 16191730

[23] Sevak L McKeigue PM Marmot MG Relationship of hyperinsulinemia to dietary intake in south Asian and European men. Am J Clin Nutr (1994) 59(5):1069–74 8172093 10.1093/ajcn/59.5.1069

[24] Lovegrove JA Lovegrove SS Lesauvage SV Brady LM Saini N Minihane AM Moderate fish-oil supplementation reverses low-platelet, long-chain n-3 polyunsaturated fatty acid status and reduces plasma triacylglycerol concentrations in British Indo-Asians. Am J Clin Nutr (2004) 79(6):974–82 15159226 10.1093/ajcn/79.6.974

[25] Donin AS Nightingale CM Owen CG Rudnicka AR McNamara MC Prynne CJ Nutritional composition of the diets of South Asian, black African-Caribbean and white European children in the United Kingdom: the Child Heart and Health Study in England (CHASE). Br J Nutr (2010) 104(2):276–85 10.1017/S000711451000070X 20230652 PMC3364483

[26] Vilaseca J Salas A Guarner F Rodríguez R Martínez M Malagelada JR Dietary fish oil reduces progression of chronic inflammatory lesions in a rat model of granulomatous colitis. Gut (1990) 31(5):539–44 10.1136/gut.31.5.539 2161781 PMC1378570

[27] Monk JM Hou TY Turk HF Weeks B Wu C McMurray DN Dietary n-3 polyunsaturated fatty acids (PUFA) decrease obesity-associated Th17 cell-mediated inflammation during colitis. PLoS One (2012) 7(11):e49739 10.1371/journal.pone.0049739 23166761 PMC3500317

[28] Andoh A Tsujikawa T Ishizuka I Araki Y Sasaki M Koyama S N-3 fatty acid-rich diet prevents early response of interleukin-6 elevation in trinitrobenzene sulfonic acid-induced enteritis. Int J Mol Med (2003) 12(5):721–5 14533000

[29] Hekmatdoost A Wu X Morampudi V Innis SM Jacobson K Dietary oils modify the host immune response and colonic tissue damage following *Citrobacter rodentium* infection in mice. Am J Physiol Gastrointest Liver Physiol (2013) 304(10):G917–28 10.1152/ajpgi.00292.2012 23518681

[30] Satish Kumar R Kanmani P Yuvaraj N Paari KA Pattukumar V Arul V Traditional Indian fermented foods: a rich source of lactic acid bacteria. Int J Food Sci Nutr (2013) 64(4):415–28 10.3109/09637486.2012.746288 23181843

[31] Ceapa C Wopereis H Rezaïki L Kleerebezem M Knol J Oozeer R Influence of fermented milk products, prebiotics and probiotics on microbiota composition and health. Best Pract Res Clin Gastroenterol (2013) 27(1):139–55 10.1016/j.bpg.2013.04.004 23768559

[32] Ananthakrishnan AN Environmental risk factors for inflammatory bowel disease. Gastroenterol Hepatol (N Y) (2013) 9(6):367–74 23935543 PMC3736793

[33] Klement E Cohen RV Boxman J Joseph A Reif S Breastfeeding and risk of inflammatory bowel disease: a systematic review with meta-analysis. Am J Clin Nutr (2004) 80(5):1342–52 15531685 10.1093/ajcn/80.5.1342

[34] Twamley K Puthussery S Harding S Baron M Macfarlane A UK-born ethnic minority women and their experiences of feeding their newborn infant. Midwifery (2011) 27(5):595–602 10.1016/j.midw.2010.06.016 21035928

[35] Innis SM Dai C Wu X Buchan AM Jacobson K Perinatal lipid nutrition alters early intestinal development and programs the response to experimental colitis in young adult rats. Am J Physiol Gastrointest Liver Physiol (2010) 299(6):G1376–85 10.1152/ajpgi.00258.2010 20864654

[36] Innis SM Polyunsaturated fatty acids in human milk: an essential role in infant development. Adv Exp Med Biol (2004) 554:27–43 10.1007/978-1-4757-4242-8_5 15384565

[37] Innis SM Essential fatty acid transfer and fetal development. Placenta (2005) 26(Suppl A):S70–5 10.1016/j.placenta.2005.01.005 15837071

[38] Jacobson K Mundra H Innis SM Intestinal responsiveness to experimental colitis in young rats is altered by maternal diet. Am J Physiol Gastrointest Liver Physiol (2005) 289(1):G13–20 10.1152/ajpgi.00459.2004 15731507

[39] Bowen L Ebrahim S De Stavola B Ness A Kinra S Bharathi AV Dietary intake and rural-urban migration in India: a cross-sectional study. PLoS One (2011) 6(6):e14822 10.1371/journal.pone.0014822 21731604 PMC3120774

[40] Ponder A Long MD A clinical review of recent findings in the epidemiology of inflammatory bowel disease. Clin Epidemiol (2013) 5:237–47 10.2147/CLEP.S33961 23922506 PMC3728155

[41] Kaplan GG Hubbard J Korzenik J Sands BE Panaccione R Ghosh S The inflammatory bowel diseases and ambient air pollution: a novel association. Am J Gastroenterol (2010) 105(11):2412–9 10.1038/ajg.2010.252 20588264 PMC3180712

[42] Ananthakrishnan AN McGinley EL Binion DG Saeian K Ambient air pollution correlates with hospitalizations for inflammatory bowel disease: an ecologic analysis. Inflamm Bowel Dis (2011) 17(5):1138–45 10.1002/ibd.21455 20806342

[43] Strachan DP Hay fever, hygiene, and household size. BMJ (1989) 299(6710):1259–60 10.1136/bmj.299.6710.1259 2513902 PMC1838109

[44] Yazdanbakhsh M Matricardi PM Parasites and the hygiene hypothesis: regulating the immune system? Clin Rev Allergy Immunol (2004) 26(1):15–24 10.1385/CRIAI:26:1:15 14755072

[45] Feillet H Bach JF Increased incidence of inflammatory bowel disease: the price of the decline of infectious burden? Curr Opin Gastroenterol (2004) 20(6):560–4 10.1097/00001574-200411000-00010 15703683

[46] Bernstein CN Rawsthorne P Cheang M Blanchard JF A population-based case control study of potential risk factors for IBD. Am J Gastroenterol (2006) 101(5):993–1002 10.1111/j.1572-0241.2006.00381.x 16696783

[47] Gent AE Hellier MD Grace RH Swarbrick ET Coggon D Inflammatory bowel disease and domestic hygiene in infancy. Lancet (1994) 343(8900):766–7 10.1016/S0140-6736(94)91841-4 7907734

[48] Wang TT Dabbas B Laperriere D Bitton AJ Soualhine H Tavera-Mendoza LE Direct and indirect induction by 1,25-dihydroxyvitamin D3 of the NOD2/CARD15-defensin beta2 innate immune pathway defective in Crohn disease. J Biol Chem (2010) 285(4):2227–31 10.1074/jbc.C109.071225 19948723 PMC2807280

[49] Lim W-C Hanauer SB Li YC Mechanisms of disease: vitamin D and inflammatory bowel disease. Nat Clin Pract Gastroenterol Hepatol (2005) 2(7):308–15 10.1038/ncpgasthep0215 16265284

[50] Pappa HM Grand RJ Gordon CM Report on the vitamin D status of adult and pediatric patients with inflammatory bowel disease and its significance for bone health and disease. Inflamm Bowel Dis (2006) 12(12):1162–74 10.1097/01.mib.0000236929.74040.b0 17119391

[51] Katz S Osteoporosis in patients with inflammatory bowel disease: risk factors, prevention, and treatment. Rev Gastroenterol Disord (2006) 6(2):63–71 16699475

[52] Fu YT Chatur N Cheong-Lee C Salh B Hypovitaminosis D in adults with inflammatory bowel disease: potential role of ethnicity. Dig Dis Sci (2012) 57(8):2144–8 10.1007/s10620-012-2130-7 22451117

[53] Froicu M Weaver V Wynn TA McDowell MA Welsh JE Cantorna MT A crucial role for the vitamin D receptor in experimental inflammatory bowel diseases. Mol Endocrinol (2003) 17(12):2386–92 10.1210/me.2003-0281 14500760

[54] Bhalla AK Amento EP Clemens TL Holick MF Krane SM Specific high-affinity receptors for 1,25-dihydroxyvitamin D3 in human peripheral blood mononuclear cells: presence in monocytes and induction in T lymphocytes following activation. J Clin Endocrinol Metab (1983) 57(6):1308–10 10.1210/jcem-57-6-1308 6313738

[55] Provvedini DM Tsoukas CD Deftos LJ Manolagas SC 1,25-Dihydroxyvitamin D3 receptors in human leukocytes. Science (1983) 221(4616):1181–3 10.1126/science.6310748 6310748

[56] Manolagas SC Provvedini DM Murray EJ Tsoukas CD Deftos LJ The antiproliferative effect of calcitriol on human peripheral blood mononuclear cells. J Clin Endocrinol Metab (1986) 63(2):394–400 10.1210/jcem-63-2-394 3013918

[57] Brennan A Katz DR Nunn JD Barker S Hewison M Fraher LJ Dendritic cells from human tissues express receptors for the immunoregulatory vitamin D3 metabolite, dihydroxycholecalciferol. Immunology (1987) 61(4):457–61 2832307 PMC1453440

[58] Veldman CM Cantorna MT DeLuca HF Expression of 1,25-dihydroxyvitamin D(3) receptor in the immune system. Arch Biochem Biophys (2000) 374(2):334–8 10.1006/abbi.1999.1605 10666315

[59] D’Ambrosio D Cippitelli M Cocciolo MG Mazzeo D Di Lucia P Lang R Inhibition of IL-12 production by 1,25-dihydroxyvitamin D3. Involvement of NF-kappaB downregulation in transcriptional repression of the p40 gene. J Clin Invest (1998) 101(1):252–62 10.1172/JCI1050 9421488 PMC508562

[60] Rigby WF Stacy T Fanger MW Inhibition of T lymphocyte mitogenesis by 1,25-dihydroxyvitamin D3 (calcitriol). J Clin Invest (1984) 74(4):1451–5 10.1172/JCI111557 6332829 PMC425314

[61] Jørgensen SP Agnholt J Glerup H Lyhne S Villadsen GE Hvas CL Clinical trial: vitamin D3 treatment in Crohn’s disease – a randomized double-blind placebo-controlled study. Aliment Pharmacol Ther (2010) 32(3):377–83 10.1111/j.1365-2036.2010.04355.x 20491740

[62] Cantorna MT Munsick C Bemiss C Mahon BD 1,25-Dihydroxycholecalciferol prevents and ameliorates symptoms of experimental murine inflammatory bowel disease. J Nutr (2000) 130(11):2648–52 11053501 10.1093/jn/130.11.2648

[63] Denson LA Long MD McGovern DP Kugathasan S Wu GD Young VB Challenges in IBD research: update on progress and prioritization of the CCFA’s research agenda. Inflamm Bowel Dis (2013) 19(4):677–82 10.1097/MIB.0b013e31828134b3 23448796 PMC3740342

[64] Ventham NT Kennedy NA Nimmo ER Satsangi J Beyond gene discovery in inflammatory bowel disease: the emerging role of epigenetics. Gastroenterology (2013) 145(2):293–308 10.1053/j.gastro.2013.05.050 23751777 PMC3919211

[65] Jostins L Ripke S Weersma RK Duerr RH McGovern DP Hui KY Host-microbe interactions have shaped the genetic architecture of inflammatory bowel disease. Nature (2012) 491(7422):119–24 10.1038/nature11582 23128233 PMC3491803

[66] Brant SR Promises, delivery, and challenges of inflammatory bowel disease risk gene discovery. Clin Gastroenterol Hepatol (2013) 11(1):22–6 10.1016/j.cgh.2012.11.001 23131344

[67] Luther J Dave M Higgins PDR Kao JY Association between *Helicobacter pylori* infection and inflammatory bowel disease: a meta-analysis and systematic review of the literature. Inflamm Bowel Dis (2010) 16(6):1077–84 10.1002/ibd.21116 19760778 PMC4865406

[68] Feeney MA Murphy F Clegg AJ Trebble TM Sharer NM Snook JA A case-control study of childhood environmental risk factors for the development of inflammatory bowel disease. Eur J Gastroenterol Hepatol (2002) 14(5):529–34 10.1097/00042737-200205000-00010 11984151

[69] Koloski NA Hygiene hypothesis in inflammatory bowel disease: a critical review of the literature. World J Gastroenterol (2008) 14(2):165 10.3748/wjg.14.165 18186549 PMC2675108

[70] Hunter MM McKay DM Review article: helminths as therapeutic agents for inflammatory bowel disease. Aliment Pharmacol Ther (2004) 19(2):167–77 10.1111/j.0269-2813.2004.01803.x 14723608

[71] Summers RW Elliott DE Urban JF Thompson RA Weinstock JV *Trichuris suis* therapy for active ulcerative colitis: a randomized controlled trial. Gastroenterology (2005) 128(4):825–32 10.1053/j.gastro.2005.01.005 15825065

[72] Cornish JA Tan E Simillis C Clark SK Teare J Tekkis PP The risk of oral contraceptives in the etiology of inflammatory bowel disease: a meta-analysis. Am J Gastroenterol (2008) 103(9):2394–400 10.1111/j.1572-0241.2008.02064.x 18684177

[73] Khalili H Higuchi LM Ananthakrishnan AN Richter JM Feskanich D Fuchs CS Oral contraceptives, reproductive factors and risk of inflammatory bowel disease. Gut (2013) 62(8):1153–9 10.1136/gutjnl-2012-302362 22619368 PMC3465475

[74] Cutolo M Capellino S Montagna P Ghiorzo P Sulli A Villaggio B Sex hormone modulation of cell growth and apoptosis of the human monocytic/macrophage cell line. Arthritis Res Ther (2005) 7(5):R1124–32 10.1186/ar1791 16207329 PMC1257440

[75] Williamson LM Parkes A Wight D Petticrew M Hart GJ Limits to modern contraceptive use among young women in developing countries: a systematic review of qualitative research. Reprod Health (2009) 6:3 10.1186/1742-4755-6-3 19228420 PMC2652437

[76] Schultsz C Van Den Berg FM Ten Kate FW Tytgat GN Dankert J The intestinal mucus layer from patients with inflammatory bowel disease harbors high numbers of bacteria compared with controls. Gastroenterology (1999) 117(5):1089–97 10.1016/S0016-5085(99)70393-8 10535871

[77] Manichanh C Rigottier-Gois L Bonnaud E Gloux K Pelletier E Frangeul L Reduced diversity of faecal microbiota in Crohn’s disease revealed by a metagenomic approach. Gut (2006) 55(2):205–11 10.1136/gut.2005.073817 16188921 PMC1856500

[78] Pils MC Bleich A Prinz I Fasnacht N Bollati-Fogolin M Schippers A Commensal gut flora reduces susceptibility to experimentally induced colitis via T-cell-derived interleukin-10. Inflamm Bowel Dis (2011) 17(10):2038–46 10.1002/ibd.21587 21182023

[79] Sellon RK Tonkonogy S Schultz M Dieleman LA Grenther W Balish E Resident enteric bacteria are necessary for development of spontaneous colitis and immune system activation in interleukin-10-deficient mice. Infect Immun (1998) 66(11):5224–31 9784526 10.1128/iai.66.11.5224-5231.1998 PMC108652

[80] Yang I Eibach D Kops F Brenneke B Woltemate S Schulze J Intestinal microbiota composition of interleukin-10 deficient C57BL/6J mice and susceptibility to *Helicobacter hepaticus*-induced colitis. PLoS One (2013) 8(8):e70783 10.1371/journal.pone.0070783 23951007 PMC3739778

[81] Card T Antibiotic use and the development of Crohn’s disease. Gut (2004) 53(2):246–50 10.1136/gut.2003.025239 14724158 PMC1774910

[82] Shaw SY Blanchard JF Bernstein CN Association between the use of antibiotics in the first year of life and pediatric inflammatory bowel disease. Am J Gastroenterol (2010) 105(12):2687–92 10.1038/ajg.2010.398 20940708

[83] Shaw SY Blanchard JF Bernstein CN Association between the use of antibiotics and new diagnoses of Crohn’s disease and ulcerative colitis. Am J Gastroenterol (2011) 106(12):2133–42 10.1038/ajg.2011.304 21912437

[84] Albenberg LG Lewis JD Wu GD Food and the gut microbiota in inflammatory bowel diseases: a critical connection. Curr Opin Gastroenterol (2012) 28(4):314–20 10.1097/MOG.0b013e328354586f 22573192 PMC3822011

[85] Lozupone CA Stombaugh JI Gordon JI Jansson JK Knight R Diversity, stability and resilience of the human gut microbiota. Nature (2012) 489(7415):220–30 10.1038/nature11550 22972295 PMC3577372

[86] De Filippo C Cavalieri D Di Paola M Ramazzotti M Poullet JB Massart S Impact of diet in shaping gut microbiota revealed by a comparative study in children from Europe and rural Africa. Proc Natl Acad Sci U S A (2010) 107(33):14691–6 10.1073/pnas.1005963107 20679230 PMC2930426

[87] Wu GD Chen J Hoffmann C Bittinger K Chen YY Keilbaugh SA Linking long-term dietary patterns with gut microbial enterotypes. Science (2011) 334(6052):105–8 10.1126/science.1208344 21885731 PMC3368382

[88] Hehemann JH Kelly AG Pudlo NA Martens EC Boraston AB Bacteria of the human gut microbiome catabolize red seaweed glycans with carbohydrate-active enzyme updates from extrinsic microbes. Proc Natl Acad Sci U S A (2012) 109(48):19786–91 10.1073/pnas.1211002109 23150581 PMC3511707

[89] Deatherage Kaiser BL Li J Sanford JA Kim YM Kronewitter SR Jones MB A multi-omic view of host-pathogen-commensal interplay in *Salmonella*-mediated intestinal infection. PLoS One (2013) 8(6):e67155 10.1371/journal.pone.0067155 23840608 PMC3694140

